# Long-Term Impacts of Early Musical Abilities on Academic Achievement: A Longitudinal Study

**DOI:** 10.3390/jintelligence10030036

**Published:** 2022-06-29

**Authors:** Márta Janurik, Krisztián Józsa

**Affiliations:** 1 Béla Bartók Faculty of Arts, University of Szeged, 6722 Szeged, Hungary; gevayne.janurik.marta@zmk.szte.hu; 2 Institute of Education, University of Szeged, 6722 Szeged, Hungary; 3 Institute of Education, Hungarian University of Agriculture and Life Sciences, 7400 Kaposvár, Hungary

**Keywords:** early musical abilities, GPA, school success, academic achievement, predictive role of musical abilities

## Abstract

Numerous neurological, psychological, and transfer studies confirmed the role of learning music in cognitive development and education. However, exploring the long-term impacts of early musical abilities on academic achievement has gained relatively little attention thus far. In a seven-year longitudinal study, we examined the predictive role of musical abilities in future success in school. The sample consisted of 76 Hungarian students. The independent variables were mothers’ education and the tests administered to Grade-1 students, which included Raven’s Progressive Matrices and tests on word reading, mathematics, and musical abilities. The dependent variable was GPA in Grade 7. All tests demonstrated adequate reliability. In the regression model with the most significant predictive role, the independent variables explained 46% of GPA in Grade 7 when taken together. We established the long-term predictive role of musical abilities in later success in school. Rhythm perception and reproduction demonstrated the most significant explanatory power (11%) of variance for GPA. Mathematics and mothers’ education each explained 10% of the variance. The findings shed light on the positive impacts that early musical training may play in later academic achievement, even in the long run.

## 1. Introduction

Many studies have confirmed that early academic skills predict later academic achievement (Duncan et al. 2007). Moreover, psychological and neuroscientific studies provide evidence of the role of music learning in cognitive development. The development of musical abilities is associated with other cognitive skills and academic achievement (Miendlarzewska and Trost 2013; Tierney and Kraus 2013a). However, longitudinal studies on the role of early musical abilities in later academic achievement are scarce. A few studies on music transfer explored the role of learning to play an instrument, which is a process accompanied by the development of complex cognitive and musical abilities in academic achievement. In these studies, music training is considered an independent variable. Furthermore, most music programs used in experiments typically include learning to play an instrument as part of skill enhancement. However, learning to play an instrument is a complex activity that may facilitate the development of several musical and nonmusical abilities. It requires persistent and focused attention, decoding of complex musical patterns and visual symbols, acquisition of musical structures (e.g., intervals, scales, and chords), continuous improvement of motor coordination, and familiarization with music styles, as well as the expression of emotions through musical content (Huttenlocher 2002). Certain cognitive skills (e.g., inhibitory control, cognitive flexibility, or working memory), indispensable to playing a musical instrument, are components of executive functions (Okada and Slevc 2020).

Furthermore, the daily practice of playing a musical instrument may enhance the development of personal characteristics, such as persistence, determination, even mastery motivation, and self-regulated learning, which are beneficial to learning and academic achievement (Józsa and D. Molnár 2013). As one may conclude, children engaged in learning to play an instrument or other music programs that include playing or improvising simple instruments encounter diverse opportunities to enhance their music and non-music skills and abilities. Moreover, examining the factors that may prompt individuals to commit to music learning is important. These factors may include two of the Big Five personality dimensions, namely, conscientiousness (includes self-discipline, organization, and achievement-orientation) and openness to experience (Corrigall et al. 2013).

However, one may assume that the impact of music learning reported in previous studies can be captured even at a simple level in childhood. At the musical perception level, the perception of musical patterns (e.g., pitch and rhythm), or at the level of musical reproduction (singing and clapping; Hargreaves and Aksentijevic 2011). Studies on the transfer effects of music training rely on experiments with different durations; however, they typically last no longer than a few months. To the best of our knowledge, no study has used a longitudinal design to examine the predictive role of early musical perception and reproduction (e.g., singing and clapping), which form the foundation of all musical abilities, in academic achievement several years later. Alternatively, the novelty of our study is its duration, which is seven years. Moreover, its relevant aspect is that we explored the role of early skills in learning and the predictive role of two variables, namely, intelligence and parents’ education, which are considered relatively fixed individual capacities.

## 2. Theoretical Framework

### 2.1. Music Learning and Academic Achievement

Previous findings have established the positive impact of music learning (instrumental learning or participation in a choir or band) on academic achievement in general as well as on domain-specific achievement among elementary school, secondary school, and university students (e.g., Babo 2004; Gouzouasis et al. 2007). Cabanac et al. (2013) revealed that secondary school students who continued with music training after completing compulsory music education performed significantly better in all school subjects. Moreover, Santos-Luiz et al. (2015) examined the academic achievement of music and non-music students from Grades 7 to 9. They found that music students exhibited higher levels of academic achievement than non-music students did. Catterall et al. (1999) established a positive relationship between arts education and academic achievement and reported that the gains for arts-involved students became more prominent over time. Students consistently involved in high levels of instrumental music learning demonstrated significantly higher levels of mathematics proficiency than unengaged students. Morrison (1994) analyzed data from the National Center for Educational Statistics, a branch of the United States Department of Education that collected information on more than 18,000 high school students. The authors found that students who participated in music learning performed better in English, mathematics, history, and science. In another large-scale study on 4739 elementary and middle school students from the United States, Johnson and Memmott (2006) reported that elementary school students who participated in high-quality music education programs scored high on English and mathematics standardized tests. Moreover, middle school students who participated in a high-quality music or low-quality instrumental programs scored high on English and mathematics standardized tests. However, the effect sizes in these studies were small.

Evidence suggests that children and adolescents perform better in the following domains in school due to music training: language arts (Babo 2004; Kinney 2008; Morrison 1994; Santos-Luiz et al. 2015; Young et al. 2014; Zanutto 1997), reading (Babo 2004; Butzlaff 2000; David et al. 2007; Moreno et al. 2009; Zanutto 1997), mathematics (Babo 2004; Cabanac et al. 2013; Cheek and Smith 1999; Gouzouasis et al. 2007; Johnson and Memmott 2006; Morrison 1994; Santos-Luiz et al. 2015; Zanutto 1997), history (Cabanac et al. 2013; Morrison 1994), physics (Cabanac et al. 2013), chemistry (Cabanac et al. 2013), geography (Santos-Luiz et al. 2015), biology (Gouzouasis et al. 2007), natural science (Santos-Luiz et al. 2015), science (Morrison 1994; Zanutto 1997), foreign languages (Santos-Luiz et al. 2015), and sports (Cabanac et al. 2013).

In exploring the impacts of music learning and understanding their underlying mechanisms, these studies have also included specific variables such as prior academic achievement (Kinney 2008), intelligence (Corrigall et al. 2013; Santos-Luiz et al. 2015; Schellenberg 2006), personality characteristics of children (Corrigall et al. 2013), socioeconomic status (SES; Babo 2004; Catterall et al. 1999; Kinney 2008; Miksza 2007; Santos-Luiz et al. 2015; Schellenberg 2006; Young et al. 2014), or nonmusical activities (Schellenberg 2006), and examined their role as well.

### 2.2. Musical and Cognitive Abilities

Although the perception of musical patterns, the capability to perform musical activities, and pleasure in music are universal abilities, significant individual differences may exist in these abilities. Music aptitude refers to natural musical abilities or an innate potential to succeed as a musician (Sloboda 1994). In a narrow sense, musical abilities are those that entirely relate to musical content. Musical perception, which relies on cognitive operations with auditory information, is the foundation of all complex musical abilities. According to the modular approach of musical perception, pitch and timing or temporal processing are distinct within the music processing module (Peretz 2009). Pitch perception is concerned with three important characteristics of musical sound patterns, namely, pitch (perception of the frequency of a sound), melody (recognition of the order of sounds), and harmony (perception of key membership, tonal hierarchy, and harmony change). Rhythm in music has two independent hierarchical organizations: grouping (entails the segmentation of small units into large and even larger units) and meter (the periodic alternation of strong and weak beats; Jackendoff and Lerdahl 2006).

Reproduction, singing melodies after listening to them, or clapping or tapping back rhythmic patterns, is also complex. Previous studies have pointed to the complex systems that underlie proficient singing and rhythm reproduction. Berkowska and Bella (2009) proposed the vocal sensorimotor loop model, where perceptual and motor planning components, memory retrieval, auditory–motor mapping, and complex feedback mechanisms are key elements of singing. Moreover, working memory, attention, motor control, coordination, and planning all play a role in the development of rhythm reproduction (Drake et al. 2000). Musical perception and reproduction develop through the years due to cognitive development and musical experience, and childhood plays a crucial role in this process (Asztalos and Csapó 2017; Dowling 2002).

Although conventional protocols can measure musical perception and reproduction, musical aptitude tests typically assess the level of development in musical perception (Gordon 1989). Such tests are based on the same–different tasks. The level of performance in these tasks, in which participants must determine whether two sequences of sounds are the same or different, is linked to the ability to recall sounds in working memory (Besson et al. 2011). In addition, Degé et al. (2015) established the significant correlation between rhythm perception and production through rapid retrieval from long-term and working memory. Law (2012) defined two perceptual strategies for processing musical information. The first pertains to using memory to store and recall auditory elements; the second denotes utilizing auditory sensory mechanisms to process auditory elements. This study demonstrated the positive relationships of rhythm-to-melody, rhythm, accent, and melody subtests with short-term memory capacities (simple span task) and working memory (complex manipulation of original information).

Previous results mainly indicate positive correlations between intelligence scores and musical ability tests; however, the correlation coefficient is typically low at approximately 0.30 (Shuter-Dyson 1999). Several studies have confirmed a link between music learning and intelligence (Esteki 2013; Schellenberg 2004, 2006, 2011). According to Helmbold et al. (2006), temporal and pitch discrimination can be considered valid predictors of psychometric intelligence. Moreover, Costa-Giomi (1999) reports the positive impact of piano instruction on the development of spatial skills. Daily music training may also enhance the development of creativity. In a four-year longitudinal study on students who received Kodály-based music education through daily music classes, Barkóczi and Pléh (1977) identified enhanced creativity.

Recent results suggest that musical activities may influence the development of executive functions. Rhythm perception and reproduction are associated with certain components of intelligence that play an important role in learning, such as working memory (Degé et al. 2015), music training, which enhances inhibitory control (Bugos and DeMarie 2017; Frischen et al. 2019; Moreno et al. 2011), and cognitive flexibility (Bugos et al. 2007; Portowitz et al. 2014). These components are executive functions. Degé et al. (2011) established a significant association between the duration of music training and intelligence, mediated by executive functions. Furthermore, Moreno et al. (2011) suggest that musical activities that are indirectly related to playing musical instruments also facilitate the development of executive functions. Previous studies on the impacts of music mainly relied on musical activities that included playing instruments. However, Janurik et al. (2019) shifted their focus to a simple level, musical perception, and established significant correlations between pitch and rhythm perception and executive functions. The highest correlation, which was moderate (*r* = 0.53), was established through moderately challenging tasks with self-regulated inhibition and executive function components that promote mental set-shifting. In these moderately challenging tasks, pitch and rhythm discrimination exhibited significant correlations; in challenging tasks, however, only rhythm perception correlated significantly with the components of executive function. Furthermore, melody and pitch perception correlated significantly with working memory. In addition, other studies point to the potential role of executive functions in developing rhythm reproduction (Tierney and Kraus 2013b).

### 2.3. Early Reading and Mathematical Skills and Early Musical Abilities

Reading and arithmetic are the two most fundamental skills, in which acquisition is the most important objective of teaching during the early school years. Specifically, reading is a complex, multi-level activity. At the first level, three cognitive abilities, namely, phonological awareness, phonics, and rapid automatized naming, play crucial roles in the early development of reading (Ziegler and Goswami 2005). Previous scholars establish that music training promotes the success of reading acquisition. The theoretical approaches of this link assume common characteristics for musical and speech perception. In other words, music may stimulate cognitive abilities, such as auditory perception, and facilitate speech perception in early childhood. The assumption that language and music share common auditory-processing mechanisms is becoming accepted (Patel 2012). The majority of available evidence of perceptual language abilities in reading acquisition is related to the relationship between phonological awareness and musical abilities (Degé and Schwarzer 2011; Holliman et al. 2010; Moreno et al. 2011). Phonological awareness is an essential precursor of later reading ability and refers to reflecting on and manipulating the sound structure of spoken words (for a review, see Melby-Lervag et al. 2012). Other perceptual language skills are connected to musical perception. Examples of these skills are prosody perception (lexical identification; Wong and Perrachione 2007), vocabulary (Moyeda et al. 2006), and verbal memory (Ho et al. 2003).

Temporal and pitch representation, rapid auditory processing, auditory working memory, and recognition of auditory patterns do not only form the basis of musical perception but also play a role in reading acquisition (Tierney and Kraus 2013a). Several studies report a positive relationship between music learning and reading in the early stages of learning to read (e.g., David et al. 2007; Holliman et al. 2010; Moreno et al. 2009), whereas other studies are unable to establish such a relationship (Lukács and Hombolygó 2019). Conversely, Butzlaff (2000) describes two meta-analyses in a review. The findings reveal that correlational studies reported a positive association between music training and reading abilities. However, experimental studies fail to establish this significant correlation. This contradiction suggests that perceptual language abilities may mediate the impacts of music training. According to the review of Schellenberg and Weiss (2013), the evidence that music training has a causal effect on reading does not seem to be strong enough; the causal effect between music lessons and phonological awareness is more compelling.

There are three levels in the early development of mathematical skills: number–word sequence isolated from quantities (basic numerical skills), quantity to number–word linkage (linking number words with quantity), and linking quantity relations with number words (concept of number relationships; Krajewski and Schneider 2009). For example, Wenger and Wenger (1990) suggested that exercising cortical neurons during musical activities may enhance the areas of the cortex that play a role in mathematical thinking. Presumably, music training activates and improves processes that connect different systems to represent natural numbers. The National Council of Teachers of Mathematics in the United States pointed out the focal points of linking music and mathematics (Kells 2008). They include numbers and operations (understanding whole numbers, concepts of correspondence, counting, cardinality, and comparison) linked to counting beats (more or less); geometry (identifying shapes and describing spatial relationships) linked to notation (high/low notes on the staff); and measurement (identifying measurable attributes and identifying objects by comparing measurable attributes) linked to tonality (high/low notes and tempo discrimination). A wide range of studies confirmed the positive impact of music learning on mathematics achievement among elementary school students (Haley 2001; Hurwitz et al. 1975; Spelke 2008; Nisbet 1991). For example, Vaughn (2000) conducted a meta-analysis on correlational studies and concluded that a small positive association exists between music training and mathematical abilities.

An increasing number of researchers assume overlaps between arithmetic and reading skills. Certain cognitive skills form the foundation for learning to read and perform arithmetic. On the one hand, the results of Durand et al. (2005) confirm previous results, in which phoneme deletion is a critical foundation for learning to read. However, the authors also found that verbal abilities predict reading and arithmetic skills to a similar extent. Other scholars confirmed the role of working memory in developing reading and mathematical skills. In a three-year longitudinal study, Krajewski and Schneider (2009) concluded that early phonological awareness and visual–spatial working memory, assessed at the age of 5 years, mediated the impact through early quantity–number competencies, which predicted mathematics achievement at Grade 3. Specifically, phonological awareness predicted basic numerical competencies but not higher numerical competencies, which may explain the moderate relationship between early literacy development and the development of mathematical competencies. Based on these findings, the authors suggested that phonological awareness is a domain-general precursor of academic achievement instead of a domain-specific precursor of only subsequent literacy development in school.

### 2.4. Social Background, School Learning, and Music Learning

Research indicates that children with low SES develop academic skills more slowly than children with high SES. Children from low-SES families gain less experience that supports the development of fundamental reading acquisition skills, such as phonological awareness, vocabulary, and oral language. Furthermore, they enter the education system with less developed arithmetic and cognitive skills (Buckingham et al. 2013; Morgan et al. 2009). Several studies have suggested that music training may be a tool for compensating for disadvantages due to SES (e.g., Babo 2004; Catterall et al. 1999; Barkóczi and Pléh 1977; Schellenberg 2006; Helmrich 2010).

Social background plays a key role in developing musical abilities in childhood, which is similar to the development of other cognitive skills. Janurik and Józsa (2013) examined the development of musical perception in preschool as well as in the early school years. The authors found no difference in musical perception among preschool children based on parents’ education. However, during the first years of school (when cognitive skills typically start to improve, which is crucial for the development of musical perception), children whose parents have elementary level of education (i.e., the first eight grades) are, on average, two years lagging behind in musical abilities. However, the role of family decreases in the long run and is replaced by peer relationships, musical activities, and music learning, which influence the development of musical perception (Gembris 2006).

## 3. Objectives

The study presents the following research questions. Can a relationship be established between musical abilities and the dimensions examined (e.g., perception, reproduction, and pitch-related and rhythm-related abilities) in Grade 1 and GPA in Grade 7? To what extent does mothers’ education predict GPA in Grade 7? What is the explanatory power of IQ? Can the predictive role of musical abilities in Grade 1 be established apart from mothers’ education and IQ? To what extent do mothers’ education, IQ, word reading, and mathematical skills predict GPA? What is the explanatory power of musical abilities? Can its predictive role be established apart from mothers’ education, IQ, word reading, and mathematical skills?

## 4. Method

### 4.1. Participants

The study recruited participants from schools located in the southern part of Hungary. We followed the development of 76 students (male: 40) from eight classes between Grades 1 and 7. The schools were situated in advantaged regions, as five were in a city with a university. All children were monolingual native speakers of Hungarian without known hearing or neurological deficits, attentional deficit disorders, or reading disabilities. The mean age of participants was 6 years and 6 months at the start of the investigation (*M* = 79.34, *SD* = 4.36 months). We used mothers’ education as the social background variable. In total, 28, 28, and 20 of the mothers achieved the elementary, secondary, and higher levels of education, respectively. The difference between the number of participants of the sub-samples based on mothers’ education is non-significant (χ^2^ = 1.684, *p* = 0.431). Moreover, 12 participants were engaged in after-school music programs. Out of them, three learned to play a musical instrument in an after-school program for one year, three for 2 years, and three for 3 years. One participant learned to play a musical instrument for 5 years, one for 6 years, and one for 8 years. The parents provided written consent to assess their children and record their data.

### 4.2. Measures

#### 4.2.1. Musical Aptitude Test

The musical aptitude test consisted of 75 items (Surján and Janurik 2018). The items can be grouped in two ways, which allowed two ways of creating subtests. The first is formulating subtests for musical perception and musical reproduction. The second is creating subtests for pitch-related abilities (pitch perception and reproduction) and rhythm-related abilities (rhythm perception and reproduction). The raw scores of musical aptitude were transformed into percentage values, range 0–100.

#### 4.2.2. Musical Perception and Musical Reproduction

In this arrangement, the musical aptitude test categorized musical skills or items based on two aspects. First, it measured the level of (1) development of musical perception using listening discrimination tasks and (2) musical reproduction using singing and clapping tasks.

The first section of the test, which measured musical perception, consisted of tape-recorded discrimination tasks (42 items). The participants had to decide whether two successive musical sound patterns were the same or different. The task types were as follows:

(1) Melody discrimination: deciding whether two successive short, sung melodies were the same or different (6 items);
(2) Chord analysis: determining the number of notes heard at once (5 items);
(3) Tempo discrimination: short, simple piano excerpts with the same or different tempo at repetition (5 items);
(4) Interval discrimination: two successive ascending/descending intervals played on the piano (7 items);
(5) Rhythm discrimination: two successive rhythmic patterns played on a snare drum (6 items);
(6) Timbre discrimination: comparing the sounds of instruments from the same or a different instrument family with a slightly similar timbre (5 items);
(7) Chord discrimination: two successive chords (6 items);
(8) Dynamics: comparing the dynamics of five-beat music excerpts played on the piano (2 items).

A 2.5-second delay was used between the music excerpts being compared.

The second section of the test consisted of three *musical reproduction* tasks (33 items), namely, rhythm-clapping (8 items), interval-singing (18 items), and melody-singing (7 items).

#### 4.2.3. Pitch-Related Abilities and Rhythm-Related Abilities

Grouping test items differently enabled the examination of pitch-related and rhythm-related abilities separately.

The items that measured pitch-related abilities were as follows (49 items):

(1) Melody discrimination: deciding whether two successive short, sung melodies were the same or different (6 items);
(2) Chord analysis: determining the number of notes heard at once (5 items);
(3) Interval discrimination: two successive ascending/descending intervals played on the piano (7 items);
(4) Chord discrimination: two successive chords (6 items);
(5) Interval-singing (18 items);
(6) Melody-singing (7 items).

The rhythm-related subtest consisted of the following tasks (19 items):

(1) Tempo discrimination: short, simple piano excerpts with the same or different tempo at repetition (5 items);
(2) Rhythm discrimination: two successive rhythmic patterns played on a snare drum (6 items);
(3) Rhythm-clapping (8 items).

The composite index of the *combined musical aptitude test* included all test items. The subtests for pitch- and rhythm-related abilities exclude dynamics and timbre discrimination tasks because these tasks are neither related to pitch perception and reproduction nor to rhythm perception and reproduction.

#### 4.2.4. Intelligence

We used Raven’s Progressive Matrices to measure non-verbal intelligence (Raven et al. 1998). The test comprises three sets of matrices, namely, A, AB, and B. The three sets are considered the three sub-scales of the test: A (11 items, Cronbach’s alpha: 0.89), AB (12 items, Cronbach’s alpha: 0.90), and B (12 items, Cronbach’s alpha: 0.88). The overall reliability for the 35 items was 0.89. The raw scores were transformed into percentage values ranging from 0 to 100.

#### 4.2.5. Word Reading

We measured the level of development in word reading in Grade 1 to assess reading ability (Nagy 2004). The criterion-oriented diagnostic test is based on the critical vocabulary of an optimal level of word reading competency. The items are related to headwords (37 items), synonyms (15 items), and word-meaning (15 items) for a total number of 67.

#### 4.2.6. Elementary Arithmetic Skills

The test was developed based on the Grade-1 standards of the Hungarian National Core Curriculum and the Principles and Standards for School Mathematics of the United States of America (National Council of Teachers of Mathematics 2000; Józsa and Kelemen 2007). It assesses the following skills: counting, reading numbers, place value, ratio, ordinal numbers, and basic arithmetic operations. The test consists of 77 items. The raw scores of the subtests were transformed into percentage values ranging from 0 to 100.

#### 4.2.7. School Marks

Data related to students’ marks and achievements were requested from the participating schools. The parents of five students did not consent to include their children’s GPAs in the study. All participants learned the same school subjects; GPA was calculated from the midterm marks in 12 subjects in Grade 7. Academic achievement and GPA in Hungary are measured using a five-point scale, with 5 representing the highest level of achievement.

### 4.3. Procedure

The musical perception test (listening discrimination) in Grade 1 was administered in groups. The students listened to audio files that contained tasks, then each student filled in their responses in writing. An examiner verbally provided the written instructions for each task. The allocated time for test completion was 40 min. The students were assessed individually in singing and rhythm clapping. First, they listened to the audio files, then reproduced the clapping and singing tasks. University colleagues helped with the test recordings. Only the examiner and one participant were present in the room per assessment. Test completion required 10 min per participant. Word reading, elementary arithmetic skills, and intelligence tests were paper–pencil tests administered during regular class hours. One test was completed per lesson.

## 5. Results

### 5.1. Descriptive Statistics

Table 1 displays the mean and standard deviation of the tests for musical abilities combined and the scores for the subtests, the level of development in arithmetic skills and intelligence, the mean and standard deviation of the GPA, and the reliability values of each test.

### 5.2. Intercorrelations of Variables

The main focus of this research was to examine the long-term impacts of early musical abilities. We examined the extent to which musical abilities (i.e., musical perception, reproduction, and pitch- and rhythm-related abilities), reading, arithmetic skills, and intelligence in Grade 1 and mothers’ education predict GPA in Grade 7. Before executing these principal analyses, the study conducted intercorrelations.

Table 2 indicates that the correlation between the combined musical abilities test and musical perception (*r* = 0.72, *p* < 0.001) and rhythm-related abilities (*r* = 0.69, *p* < 0.001) is moderate/strong; moreover, the correlation between reproduction (*r* = 0.91, *p* < 0.001) and pitch-related abilities (*r* = 0.96, *p* < 0.001) is strong. These strong relationships are due to the fact that these variables are dependent on one another, that is, they share common items in varying proportions. Musical perception displayed a moderate/weak (*r* = 0.37, *p* = 0.001) correlation with reproduction and a moderate correlation with rhythm-related abilities (*r* = 0.49, *p* < 0.001).

Mothers’ education exhibited a significant, moderate correlation with the combined musical abilities test (*r* = 0.28, *p* = 0.014) as well as with musical perception (*r* = 0.31, *p* = 0.006) and pitch-related abilities (*r* = 0.31, *p* = 0.007). We established significant, moderate to medium (0.33–0.48) correlations between musical abilities in Grade 1 and learning musical instruments in later years. Learning to play a musical instrument did not correlate significantly with the other independent variables.

Correlation between arithmetic skills and word reading was found to be moderate (*r* = 0.37, *p* < 0.001). These two basic skills did not correlate significantly with mothers’ education. The correlation between arithmetic skills and the combined musical abilities test was *r* = 0.25 (*p* = 0.031), whereas the correlations between arithmetic skills and musical subtests were low to moderate (0.19–0.31). There was no significant correlation between arithmetic skills and pitch-related abilities. Word reading displayed significant, low correlations with the combined musical abilities test (*r* = 0.23, *p* < 0.044) and musical perception (*r* = 0.23, *p* < 0.050). Alternatively, its correlation with rhythm-related abilities was found to be moderate (*r* = 0.34, *p* = 0.002).

Intelligence in Grade 1 exhibited the same, moderate correlation with arithmetic skills and GPA in Grade 7 (*r* = 0.46, *p* < 0.001). Moreover, its correlation with mothers’ education was *r* = 0.48 (*p* < 0.001), whereas no significant correlation was established with later instrumental training. We found a significant, low correlation between intelligence and the combined musical abilities test (*r* = 0.24, *p* < 0.038). Furthermore, significant correlations were established between pitch-related abilities (*r* = 0.26, *p* < 0.026) and musical perception (*r* = 0.27, *p* < 0.019).

All independent variables exhibited significant, moderate correlations (0.29–0.47) with GPA in Grade 7, except for instrumental learning. The correlation between GPA and the combined musical abilities test was *r* = 0.38 (*p* = 0.001). In addition, GPA demonstrated moderate correlations with the subtests for musical abilities: the correlations with rhythm-related abilities was *r* = 0.43 (*p* < 0.001) (musical perception: *r* = 0.30, *p* = 0.010; reproduction: *r* = 0.32, *p* = 0.007; and pitch-related abilities: *r* = 0.29, *p* = 0.015). Further moderate correlations were established between GPA and word reading (*r* = 0.46, *p* < 0.001), arithmetic skills (*r* = 0.47, *p* < 0.001), intelligence (*r* = 0.47, *p* < 0.001), and mothers’ education (*r* = 0.41, *p* < 0.001).

### 5.3. Regression Models

We ran hierarchical, linear regression models to establish mothers’ education and intelligence’s explanatory power and musical abilities on GPA in Grade 7. Given that after-school instrumental training did not significantly correlate with GPA, we excluded this variable from further analyses.

#### 5.3.1. Regression Model I

We tested the following models using step-by-step regression: the dependent variable was GPA in Grade 7. Mothers’ education was entered as an independent variable in the first step, whereas IQ was entered in the second step. In the third step, we ran three models simultaneously using various variables, which we further referred to as models 3A, 3B, and 3C. In model 3A, musical abilities combined was entered in the third step. In model 3B, musical perception and musical reproduction were entered in the third step, while in model 3C, rhythm-related and pitch-related abilities were entered in the third step (Table 3).

In the first model, mothers’ education produced a significant 17% explanatory power (*R*^2^ = 0.17) on GPA in Grade 7 (*F*(1, 69) = 14.02, *β* = 0.41, *t*(1, 69) = 3.77, *p* < 0.001). In Step 2, intelligence added a further 10% increase in explained variance (*R*^2^ = 0.27). Lastly, intelligence demonstrated a 16% explanatory power (*rβ* = 0.16, *β* = 0.35, *t*(2, 68) = 2.97, *p* = 0.004), whereas mothers’ education reached 10% in this models (*β* = 0.25, *t*(2, 68) = 2.11, *p* = 0.038).

In Step 3A, adding musical abilities combined increased the explanatory power of the model by 6%, where explained variance reached 33% (*R*^2^ = 0.33). Intelligence, which demonstrated a 15% explanatory power in GPA, was found to be the most significant variable (*β* = 0.33, *t*(3, 67) = 2.88, *p* = 0.005) in this model. Musical abilities combined demonstrated a significant 10% explanatory power (*β* = 0.26, *t*(3, 67) = 2.45, *p* = 0.017), while the predictive role of mothers’ education was found to be non-significant.

In Step 3B, adding musical perception and musical reproduction increased the explanatory power of the model and explained variance again reached 33% (*R*^2^ = 0.33). With its 15% explanatory power, intelligence remained the most significant variable (*β* = 0.33, *t*(4, 66) = 2.87, *p* = 0.005). Musical reproduction demonstrated a near-significant explanatory power (*rβ* = 0.07, *β* = 0.21, *t*(4, 66) = 1.94, *p* = 0.057), whereas the role of musical perception was non-significant in this model (*rβ* = 0.03, *β* = 0.09, *t*(4, 66) = 0.81, *p* = 0.423). In this model, mothers’ education (*rβ* = 0.08, *β* = 0.19, *t*(4, 66) = 1.59, *p* = 0.117) and musical perception (*rβ* = 0.03, *β* = 0.09, *t*(4, 66) = 0.81, *p* = 0.423) did not exert a significant explanatory power.

In Step 3C, adding rhythm- and pitch-related abilities further increased the explained variance of the independent variables by 12% (*rβ* = 0.12) compared to models 3A and 3B (*R*^2^ = 0.39). In this model, rhythm-related abilities demonstrated the most significant explanatory power at 16% (*β* = 0.37, *t*(4, 66) = 3.30, *p* = 0.002), where pitch-related abilities did not play a significant role (*rβ* = −0.01, *β* = −0.05, *t*(4, 66) = −0.039, *p* = 0.697). Intelligence demonstrated a lower explanatory power than those in previous models (*rβ* = 0.14, *β* = 0.31, *t*(4, 66) = 2.80, *p* = 0.007), whereas mothers’ education demonstrated a significant explanatory power at 9% (*rβ* = 0.09, *β* = 0.23, *t*(4, 66) = 2.00, *p* = 0.050), which is in contrast with that for model 3A.

#### 5.3.2. Regression Model II

We added word reading and mathematical skills as the independent variables in a few models, and GPA remained the dependent variable. The mother’s education, IQ, word reading, and mathematical skills were entered as the independent variables in the first step. In the second step, we ran three simultaneous models, which we further referred to as models 2A, 2B, and 2C. In model 2A, the combined musical abilities test was entered in the first step. In model 2B, musical perception and reproduction were entered in the second step. Lastly, in model 2C, rhythm-related and pitch-related abilities were entered in the second step (Table 4).

According to the first model, word reading and mathematical skills further increased the explained variance of mothers’ education and IQ by 14% (cf. Table 3; Step 2). The overall model expressed a 41% explanatory power (*R*^2^ = 0.41). Word reading and arithmetic skills demonstrated a 12% explanatory power (*rβ* = 0.12, *β* = 0.26, *t*(4, 66) = 2.40, *p* = 0.017 and *rβ* = 0.12, *β* = 0.25, *t*(4, 66) = 2.25, *p* = 0.027, respectively). Mothers’ education provided a 10% explanatory power (*rβ* = 0.10, *β* = 0.24, *t*(4, 66) = 2.23, *p* = 0.029), and intelligence produced an 8% (*rβ* = 0.08), which was found to be non-significant in this model (*β* = 0.16, *t*(4, 66) = 1.34, *p* = 0.184).

Entering musical abilities combined in Step 2A further increased the explanatory power of the independent variables in Step 1 by 2% (*R*^2^ = 0.43). The explanatory power of musical abilities combined (*rβ* = 0.06, *β* = 0.16, *t*(5, 65) = 1.57, *p* = 0.120) and intelligence (*rβ* = 0.08, *β* = 0.17, *t*(5, 65) = 1.42, *p* = 0.162) were non-significant in this model. Based on the independent variables, mathematical skills and word reading demonstrated a significant explanatory power at 11% (*rβ* = 0.11, *β* = 0.23, *t*(5, 65) = 2.05, *p* = 0.045) and 10% (*rβ* = 0.10, *β* = 0.22, *t*(5, 65) = 2.11, *p* = 0.038), respectively, whereas mothers’ education displayed a near-significant explanatory power (*rβ* = 0.08, *β* = 0.20, *t*(5, 65) = 1.85, *p* = 0.068).

Entering musical perception and musical reproduction in Step 2B once again further increased the explanatory power of the independent variables in Step 1 by 2% (*R*^2^ = 0.43). Arithmetic skills (*rβ* = 0.11, *β* = 0.23, *t*(6, 64) = 2.04, *p* = 0.046) and word reading (*rβ* = 0.10, *β* = 0.22, *t*(6, 64) = 2.11, *p* = 0.039) demonstrated the most significant explanatory power in this model. Mothers’ education represented a near-significant explanatory power at 9% (*rβ* = 0.09, *β* = 0.21, *t*(6, 64) = 1.87, *p* = 0.066). Neither intelligence (*rβ* = 0.08, *β* = 0.17, *t*(6, 64) = 1.43, *p* = 0.158) nor musical perception (*rβ* = 0.01, *β* = 0.04, *t*(6, 64) = 0.34, *p* = 0.735) or musical reproduction (*rβ* = 0.05, *β* = 0.15, *t*(6, 64) = 1.41, *p* = 0.163) were found to have significant explanatory power.

Entering rhythm- and pitch-related abilities in Step 2C further increased the significant explanatory power of the independent variables in Step 1 by 5%; explained variance was 46% in this model (*R*^2^ = 0.46). The most significant explanatory power was demonstrated by rhythm-related abilities (*rβ* = 0.11, *β* = 0.26, *t*(6, 64) = 2.23, *p* = 0.029) in this model, whereas pitch–rhythm-related abilities did not exert a significant explanatory power (*β* = −0.04, *t*(6, 64) = −3.34, *p* = 0.732). In addition to rhythm-related abilities, mothers’ education also demonstrated a significant explanatory power at 10% (*rβ* = 0.10, *β* = 0.23, *t*(6, 64) = 2.14, *p* = 0.036). Mathematical skills represented a near-significant explanatory power at 10% (*rβ* = 0.10, *β* = 0.22, *t*(6, 64) = 1.93, *p* = 0.058). However, neither intelligence (*rβ* = 0.08, *β* = 0.17, *t*(6, 64) = 1.48, *p* = 0.145) nor word reading (*rβ* = 0.08, *β* = 0.18, *t*(6, 64) = 1.63, *p* = 0.109) was found to have significant explanatory power.

## 6. Discussion

The results confirmed that early musical abilities predict GPA in Grade 7. These results are consistent with previous studies that established the positive role of learning to play a musical instrument during the school years in academic achievement (e.g., Cabanac et al. 2013; Gouzouasis et al. 2007; Santos-Luiz et al. 2015). In contrast to the complex impact of learning to play a musical instrument on music and non-music skills, this study focused on more basic skills. It proved the long-term impact of early rhythm-related (rhythm perception and rhythm reproduction) abilities on academic achievement.

All musical subtests under investigation and the combined musical abilities test correlated significantly with GPA in Grade 7. Furthermore, we established moderate correlations between GPA and early musical abilities, specifically between GPA and rhythm-related abilities (rhythm perception and reproduction).

According to the first model of regression model II, mothers’ education, word reading, and arithmetic skills in Grade 1 represented significant explanatory power in GPA in Grade 7. In model 2B, we entered pitch-related and rhythm-related abilities and found that the independent variables explained nearly 50% of the variance for GPA. That is, they played a long-term role in academic achievement. Rhythm-related abilities were the most significant variables in predicting academic achievement. In addition, mothers’ education also demonstrated a significant role. This is in line with previous results focusing on music participation (e.g., Babo 2004; Miksza 2007; Schellenberg 2006; Young et al. 2014). For example, Santos-Luiz et al. (2015) analyzed the academic achievement of 110 adolescents in a longitudinal study. Data were collected at two time points, once when the students attended Grade 7 and 3 years after, when they attended Grade 9. According to the results, the academic achievement of music students is higher than those of non-music students in Grades 7 and 9. Similar to the current results, this positive association between music training and academic achievement was held even after controlling for intelligence, SES, and motivation. Janurik (2009) focused on GPA among sixth and seventh graders and reported that students learning to play a musical instrument for a minimum of 4 years displayed significantly higher GPAs than non-music students. The explanatory power of instrumental learning was significant even after controlling for SES. In the current study, learning to play a musical instrument did not significantly affect GPA. Nevertheless, this finding is important given that the number of students who engaged in instrumental learning was relatively low in our sample. The majority of these students did so for only a short time. Thus, we assume that our findings on instrumental learning are less relevant to the present research.

The role of intelligence was not any more significant in the regression models that included all independent variables. Furthermore, word reading did not play a significant role in the model where explained variance was the highest of all regression models and included rhythm-related abilities. Based on previous findings, we may assume that this finding is due to the association between musical perception and reproduction (primarily temporal processing) in Grade 1 as well as the early development of other reading and intelligence-related cognitive skills. The explanatory power of these general skills in this domain may be represented in the explained variance demonstrated by rhythm-related abilities in this model.

Scholars provided evidence of functional overlap in brain structures involved in music and speech processing (McMullen and Saffran 2004; Patel 2012). As established in this study, these common auditory-processing mechanisms may partially explain the more significant role of early rhythm-related abilities than that of early reading. The advantage of children with more developed musical perception abilities may be reflected in the level of speech perception through more developed phonological abilities (Degé and Schwarzer 2011; Holliman et al. 2010; Moreno et al. 2009). A wide range of transfer research in the reading domain supports this idea because these studies also confirmed the positive impact of rhythm-related abilities (e.g., David et al. 2007; Overy et al. 2003).

The current findings concerning the more significant role of rhythm perception and reproduction than that of intelligence are consistent with previous studies that established the link between music participation and intelligence (Helmbold et al. 2006; Schellenberg 2011). Schellenberg (2006) conducted correlational studies on the relationship between music learning, intelligence, and academic achievement and reported that music learning in childhood was a significant predictor of intelligence in young adulthood and academic ability in high school. In his 6- to 11-year-old students, music lessons were positively associated with academic achievement and measures of intelligence, and nonmusical after-school activities were not associated with intelligence or academic achievement.

A wide range of cognitive skills related to intelligence play a role in the development of musical perception and reproduction abilities. In our model, their contribution to academic achievement may be represented by the predictive role of rhythm perception and reproduction, which they “took away from” intelligence. In his research, Thackray (1969) confirmed that a conscious analysis of certain cognitive skills, such as understanding and memorizing rhythmic structures and holistically perceiving them, are fundamental to rhythm perception. Furthermore, the development of rhythm reproduction is linked to specific cognitive skills such as working memory, attention, motor control or coordination, and planning (Drake et al. 2000).

In addition, the set of cognitive skills that previous studies identified as executive functions (working memory, inhibitory control, and cognitive flexibility) are associated with musical cognition and cognition in general, which may explain the impact of music learning on intelligence (Degé et al. 2011; Frischen et al. 2019; Holochwost et al. 2017; Jaschke et al. 2018). Achievement in musical aptitude tests, which assess rhythm and pitch perception, has also been linked to working memory, inhibitory control, and cognitive flexibility (Janurik et al. 2019). For example, Leisuk (2015) reported significant differences in children’s duration and rhythm perception ability with and without executive dysfunction. Thus, executive functions may also play a role in developing rhythm reproduction (Tierney and Kraus 2013b). In their review, Miendlarzewska and Trost (2013) emphasized the mechanism of rhythmic entrainment, which may support learning and the development of executive functions. The current study may provide further indirect evidence to support this idea. Conversely, our results indicate that musical abilities play a more significant predictive role than intelligence. They are important, especially because intelligence possesses well-defined genetic, cultural, and environmental components that determine and limit, to a certain extent, the level of learning and cognition (Neisser et al. 1996). Moreover, musical abilities can improve during early childhood even within a short time (Janurik and Józsa 2012). The results demonstrate that early cognitive and psychomotor skills linked to rhythm perception and reproduction may be associated with specific cognitive skills that influence academic achievement. Children with better rhythmic skills may perform better in acquiring literacy skills, which may lead to positive gains in academic achievement.

In Step 2B of our regression model, early arithmetic skills played a significant predictive role in individual differences in terms of GPA. This role was more significant than intelligence and reading; however, its role is less than that of rhythm-related abilities. Early arithmetic skills are strongly associated with non-verbal intelligence (Jenks et al. 2009), working memory, and executive functions (Bull et al. 2008). Moreover, many studies have noted the possible links between music and mathematical processing (Nisbet 1991; Spelke 2008; Wenger and Wenger 1990). According to a previous study on first-grade elementary school students in Hungary, the predictive roles of musical perception and arithmetic skills are similar to that of general intelligence (Gévayné 2010). In the current study, harmony discrimination and rhythm discrimination together explained 16% of the variance for academic achievement, similar to the role of arithmetic skills. Meanwhile, the explanatory power of intelligence as measured using Raven’s Progressive Matrices was higher by only 1% (Gévayné 2010).

Our results provide further evidence for the associations between early arithmetic and reading skills and the components of intelligence that play an important role in academic achievement. These abilities predicted GPA in Grade 7 to a similar and significant extent in our regression models, where the combined musical abilities tests and musical perception and reproduction were the independent variables. An increasing number of researchers assume overlaps between arithmetic and reading skills. In other words, underlying mechanisms seemingly facilitate one another and simultaneously develop arithmetic and reading skills (Durand et al. 2005; Krajewski and Schneider 2009). Several studies found substantial intercorrelations between literacy and mathematical competencies in school that range from *r* = 0.4 to *r* = 0.6 (e.g., Berg 2008; Schneider and Näslund 1999). The current study established similar correlations.

## 7. Conclusions

This longitudinal study suggests that musical abilities, especially rhythm-related abilities, as well as early arithmetic skills and mothers’ education are key predictors of GPA in Grade 7. Findings also highlight the prominent role of early reading and intelligence. Moreover, our results confirmed the moderate relationship between early arithmetic skills and early reading skills that were found by some previous studies. Indirectly, these results imply the existence of domain-general abilities used for early music, speech, and mathematical processing. The findings suggest that the development of basic skills that underlie musical perception, intelligence, and academic achievement may be linked even at a more simple level than learning to play a musical instrument at the age of 6 years. Thus, we conclude that musical abilities, primarily rhythm perception and reproduction, and early arithmetic skills in Grade 1 are associated with the development of key cognitive skills that play a role in later academic achievement. However, this aspect requires a specific examination by future studies.

Based on the independent variables under investigation, rhythm-related abilities (perception and reproduction abilities) were found to play the most significant predictive role. Thus, this study emphasizes the benefits of early music training, especially rhythmic training, for all children in the early years and, specifically, for disadvantaged children. The powerful enhancement of musical abilities in preschool and the early years of school may facilitate the development of core skills that, in turn, positively influence academic achievement in the long run.

## Figures and Tables

**Table 1:** Descriptive statistics.

Tests and Subtests	Number of Items	Cronbach’s α	Min–Max	Mean	SD
Musical abilities combined	75	0.89	0–100	42.63	14.11
Musical perception	42	0.70	0–100	57.96	12.23
Musical reproduction	33	0.93	0–100	26.53	21.63
Pitch-related abilities	49	0.88	0–100	37.62	15.18
Rhythm-related abilities	19	0.71	0–100	55.92	20.33
Raven IQ	35	0.89	0–100	78.03	16.89
Word reading	67	0.81	0–100	75.15	11.31
Mathematics	77	0.92	0–100	65.82	14.09
GPA	12	0.92	1–5	3.98	0.71

**Table 2:** Intercorrelations of variables.

Variables	1	2	3	4	5	6	7	8	9	10	11
1. Musical perception	–										
2. Musical reproduction	0.37	–									
3. Pitch-related abilities	0.71	0.86	–								
4. Rhythm-related abilities	0.39	0.69	0.49	–							
5. Musical abilities combined	0.72	0.91	0.96	0.69	–						
6. Arithmetic skills	0.23	0.19	0.19	0.31	0.25	–					
7. Word reading	0.23	0.18	0.16	0.34	0.23	0.37	–				
8. Raven IQ	0.27	0.16	0.26	0.19	0.24	0.46	0.25	–			
9. Mothers’ education	0.31	0.19	0.31	0.17	0.28	0.18	0.18	0.48	–		
10. Instrumental training	0.38	0.42	0.48	0.33	0.49	0.11	0.15	0.16	0.20	–	
11. GPA	0.30	0.32	0.29	0.43	0.38	0.47	0.46	0.47	0.41	0.19	–

Note: For *r* > 0.22, the level of significance was set at *p* <0.05; for *r* > 0.30, the level of significance was set at *p* < 0.01; and for *r* > 0.37, the level of significance was set at *p* < 0.001.

**Table 3:** Hierarchical regression models with GPA as the dependent variable.

Regression Models	*R* ^2^	*β*	*R* ^2^ *Change*	*F*	*p*
Step1					
Mothers’ education	0.17	0.41	0.17	14.19	<0.001
Step 2					
Mothers’ education		0.25			
Raven IQ	0.27	0.35	0.10	12.28	<0.001
Step 3A					
Mothers’ education		0.19			
Raven IQ		0.33			
Musical abilities combined	0.33	0.26	0.06	10.80	<0.001
Step 3B					
Mothers’ education		0.19			
Raven IQ		0.33			
Musical perception		0.09			
Musical reproduction	0.33	0.21	0.06	8.00	<0.001
Step 3C					
Mothers’ education		0.23			
Raven IQ		0.31			
Rhythm-related abilities		0.37			
Pitch-related abilities	0.39	−0.05	0.12	10.37	<0.001

**Table 4:** Hierarchical regression models with GPA as the dependent variable.

Regression Models	*R* ^2^	*β*	*R* ^2^ *Change*	*F*	*p*
Step 1					
Mothers’ education		0.24			
Raven IQ		0.16			
Word reading		0.26			
Mathematical skills	0.41	0.25	0.41	11.47	<0.001
Step 2A					
Mothers’ education		0.20			
Raven IQ		0.17			
Word reading		0.22			
Mathematical skills		0.23			
Musical abilities combined	0.43	0.16	0.02	9.88	<0.001
Step 2B					
Mothers’ education		0.21			
Raven IQ		0.17			
Word reading		0.22			
Mathematical skills		0.23			
Musical perception		0.04			
Musical reproduction	0.43	0.15	0.00	8.15	<0.001
Step 2C					
Mothers’ education		0.23			
Raven IQ		0.17			
Word reading		0.18			
Mathematical skills		0.22			
Rhythm-related abilities		0.26			
Pitch-related abilities	46	−0.04	0.03	9.01	<0.001

## Data Availability

Data are available upon request due to privacy restrictions.

## References

[B1-jintelligence-10-00036] Asztalos Kata, Csapó Benő (2017). Development of musical abilities: Cross-sectional computerbased assessments in educational contexts. Psychology of Music.

[B2-jintelligence-10-00036] Babo Gerard D. (2004). The relationship between instrumental music participation and standardized assessment achievement of middle school students. Research Music in Music Education.

[B3-jintelligence-10-00036] Barkóczi Ilona, Pléh Csaba (1977). Kodály Zenei Nevelési Módszerének Pszichológiai Hatásvizsgálata [An Examination of the Efficiency of Kodály’s Music Educational Method].

[B4-jintelligence-10-00036] Berg Derek H. (2008). Working memory and arithmetic calculation in children: The contributory roles of processing speed, short-term memory, and reading. Journal of Experimental Child Psychology.

[B5-jintelligence-10-00036] Berkowska Magdalena, Bella Simone D. (2009). Acquired and congenital disorders of sung performance: A review. Advances in Cognitive Psychology.

[B6-jintelligence-10-00036] Besson Mireille, Chobert Julie, Marie Céline (2011). Transfer of training between music and speech: Common processing, attention, and memory. Frontiers in Psychology.

[B7-jintelligence-10-00036] Buckingham Jennifer, Wheldall Kevin, Beaman-Wheldall Robyn (2013). Why poor children are more likely to become poor readers: The school years. Australian Journal of Education.

[B8-jintelligence-10-00036] Bugos Jennifer A., DeMarie Darlene (2017). The effects of a short-term music program on preschool children’s executive functions. Psychology of Music.

[B9-jintelligence-10-00036] Bugos Jennifer A., Perlstein William M., McCrae Christina S., Brophy Timothy. S., Bedenbaugh Purvis H. (2007). Individualized piano instruction enhances executive functioning and working memory in older adults. Aging & Mental Health.

[B10-jintelligence-10-00036] Bull Rebecca, Espy Kimberly A., Wiebe Sandra A. (2008). Short-term memory, working memory, and executive functioning in pre-schoolers: Longitudinal predictors of mathematical achievement at age 7 years. Journal of Developmental Neuropsychology.

[B11-jintelligence-10-00036] Butzlaff Ron (2000). Can music be used to teach reading?. Journal of Aesthetic Education.

[B12-jintelligence-10-00036] Cabanac Arnaud, Perlovsky Leonid, Bonniot-Cabanac Mary-Claude, Cabanac Michel (2013). Music and academic performance. Behavioural Brain Research.

[B13-jintelligence-10-00036] Catterall James S., Chapleau Richard, Iwanaga John, Fiske Edward B. (1999). Involvement in the arts and human development: General involvement and intensive involvement in music and theatre arts. Champions of Change: The Impact of the Arts on Learning.

[B14-jintelligence-10-00036] Cheek Joyce M., Smith Lyle R. (1999). Music training and mathematics achievement. Adolescence.

[B15-jintelligence-10-00036] Corrigall Kathleen A., Schellenberg E. Glenn, Misura Nicole M. (2013). Music training, cognition and personality. Frontiers in Psychology.

[B16-jintelligence-10-00036] Costa-Giomi Eugenia (1999). The effect of three years of piano instruction on children’s cognitive development. Journal of Research in Music Education.

[B17-jintelligence-10-00036] David Dana, Wade-Woolley Lesly, Kirby John R., Smithrim Katharine (2007). Rhythm and reading development in school-age children: A longitudinal study. Journal of Research in Reading.

[B18-jintelligence-10-00036] Degé Franziska, Schwarzer Gudrun (2011). The effect of a music program on phonological awareness in preschoolers. Frontiers in Psychology.

[B19-jintelligence-10-00036] Degé Franziska, Kubicek Claudia, Schwarzer Gudrun (2011). Music lessons and intelligence: A relation mediated by executive functions. Music Perception.

[B20-jintelligence-10-00036] Degé Franziska, Kubicek Claudia, Schwarzer Gudrun (2015). Associations between musical abilities and precursors of reading in preschool aged children. Frontiers in Psychology.

[B21-jintelligence-10-00036] Dowling W. Jay, Levitin Daniel J. (2002). The Development of music perception and cognition. Foundations of Cognitive Psychology: Core Readings.

[B22-jintelligence-10-00036] Drake Carolyn, Jones Mari Riess, Baruch Clarisse (2000). The development of rhythmic attending in auditory sequences: Attunement, referent period, focal attending. Cognition.

[B23-jintelligence-10-00036] Duncan Greg J., Dowsett Chantelle J., Claessens Amy, Magnuson Katherine, Huston Aletha C., Klebanov Pamela, Pagani Linda, Feinstein Leon, Engel Mimi, Brooks-Gunn Jeanne (2007). School readiness and later achievement. Developmental Psychology.

[B24-jintelligence-10-00036] Durand Marianne, Hulme Charles, Larkin Rebecca, Snowling Margaret (2005). The cognitive foundations of reading and arithmetic skills in 7- to 10-year-olds. Journal Experimental Child Psychology.

[B25-jintelligence-10-00036] Esteki Mahnaz (2013). Effectiveness of “Music Training” on reorganization of brain and poor intellectual abilities in female students with dyscalculia (7-9 years old). Global Journal of Arts Education.

[B26-jintelligence-10-00036] Frischen Ulrike, Schwarzer Gudrun, Degé Franziska (2019). Comparing the effects of rhythm-based music training and pitch-based music training on executive functions in preschoolers. Frontiers in Integrative Neuroscience.

[B27-jintelligence-10-00036] Gembris Heiner, Colwell R. (2006). The development of musical abilities. MENC Handbook of Musical Cognition.

[B28-jintelligence-10-00036] Gévayné Janurik Márta (2010). A Zenei Hallási Képességek Fejlődése és Összefüggése Néhány Alapkészséggel 4–8 Éves Kor Között [Development of Music Listening Skills and Their Relationship to Some Basic Learning Skills between the Ages of 4 and 8]. Ph.D. dissertation.

[B29-jintelligence-10-00036] Gordon Edwin E. (1989). Advanced Measures of Music Audiation.

[B30-jintelligence-10-00036] Gouzouasis Peter, Guhn Martin, Kishor Nand (2007). The predictive relationship between achievement and participation in music and achievement in core Grade 12 academic subjects. Music Education Research.

[B31-jintelligence-10-00036] Haley Jennifer A. (2001). The Relationship between Instrumental Music Instruction and Academic Achievement in Fourth Grade Students. Doctoral dissertation, Pace University, New York, NY, USA. Dissertation Abstracts International.

[B32-jintelligence-10-00036] Hargreaves David J., Aksentijevic Alexandar (2011). Music, IQ, and the executive function. British Journal of Psychology.

[B33-jintelligence-10-00036] Helmbold Nadin, Troche Stefan, Rammsayer Thomas (2006). Temporal information processing and pitch discrimination as predictors of general intelligence. Canadian Journal of Experymental Psychology.

[B34-jintelligence-10-00036] Helmrich Barbara H. (2010). Window of opportunity? Adolescence, music, and algebra. Journal of Adolescent Research.

[B35-jintelligence-10-00036] Ho Yim-Chi, Cheung Mei-Chun, Chan Agnes S. (2003). Music training improves verbal but not visual memory cross-sectional and longitudinal explorations in children. Neuropsychology.

[B36-jintelligence-10-00036] Holochwost Steven J., Propper Cathy B., Wolf Dennis, Willoughby Michael T., Fisher Kelly R., Kolacz Jacek, Volpe Vanessa V., Jaffee Sara (2017). Music education academic achievement, and executive functions. Psychology of Aesthetics Creativity, and the Arts.

[B37-jintelligence-10-00036] Hurwitz Irving, Wolff Peter H., Bortnick Barrie D., Kokas Klára (1975). Non-musical effects of the Kodaly music curriculum in primary grade children. Journal of Learning Disabilities.

[B38-jintelligence-10-00036] Huttenlocher Peter R. (2002). Neural Plasticity. The Effects of Environment on the Development of the Celebral Cortex.

[B39-jintelligence-10-00036] Jackendoff Ray, Lerdahl Fred (2006). The capacity for music: What is it and what’s special about it?. Cognition.

[B40-jintelligence-10-00036] Janurik Márta (2009). Hogyan viszonyulnak az általános és középiskolás tanulók a klasszikus zenéhez? [How do elementary and secondary school students feel about classical music?]. Új Pedagógiai Szemle.

[B41-jintelligence-10-00036] Janurik Márta, Józsa Krisztián (2012). Findings of a three months long music training programme. Hungarian Educational Research Journal.

[B42-jintelligence-10-00036] Janurik Márta, Józsa Krisztián (2013). A zenei képességek fejlődése négy- és nyolcéves kor között. [Development of musical abilities between ages 4 and 8.]. Magyar Pedagógia.

[B43-jintelligence-10-00036] Janurik Márta, Szabó Norbert, Józsa Krisztián, Chova Luis Gómez, Martínez Augustin López, Torres Ignazio Candel (2019). The relationship of musical perception and the executive function among 7-year-old children. EDULEARN19 Proceedings: 11th International Conference on Education and New Learning Technologies.

[B44-jintelligence-10-00036] Jaschke Artur C., Honing Henkjan, Scherder Erik J. A. (2018). Longitudinal analysis of music education on executive functions in primary school children. Frontiers in Neuroscience.

[B45-jintelligence-10-00036] Jenks Kathleen M., van Lieshout Ernest C. D. M., Moor Jan de (2009). The relationship between medical impairments and arithmetic development in children with cerebral palsy. Journal of Child Neurology.

[B46-jintelligence-10-00036] Johnson Christopher. M., Memmott Jenny E. (2006). Examination of relationships between music programes of differing quality and standardised test results. Journal of Research in Music Education.

[B47-jintelligence-10-00036] Józsa Krisztián, Molnár Éva D., Barrett Karen Caplovitz, Fox Nathan A., Morgan George A., Fidler Deborah J., Daunhauer Lisa A. (2013). The relationship between mastery motivation, self-regulated learning and school success: A Hungarian and wider European perspective. Handbook of Self-Regulatory Processes in Development: New Directions and International Perspectives.

[B48-jintelligence-10-00036] Józsa Krisztián, Kelemen Rita, Csapó Benő, Csíkos Csaba (2007). The development of elementary math: Results form a large scale longitudinal study. 12th European Conference for Research on Learning and Instruction: Developing Potentials for Learning.

[B49-jintelligence-10-00036] Kells Deanne (2008). The Impact of Music on Mathematics Achievement. https://media2.kindermusik.com/website/2016/11/Research_Schools_Kindermusik_Impact-of-MM-on-SocialEmotionalDevelopment_2016.pdf.

[B50-jintelligence-10-00036] Kinney Daryl V. (2008). Selected demographic variables, school music participation, and achievement test scores of urban middle school students. Journal of Research of Music Education.

[B51-jintelligence-10-00036] Krajewski Kristin, Schneider Wolfgang (2009). Exploring the impact of phonological awareness, visual-spatial working memory, and preschool quantity-number competencies on mathematics achievement in elementary school: Findings from a 3-year- longitudinal study. Journal of Experimental Child Psychology.

[B52-jintelligence-10-00036] Leisuk Teresa (2015). Music perception ability of children with executive function deficits. Psychology of Music.

[B53-jintelligence-10-00036] Lukács Borbála, Hombolygó Ferenc (2019). Task-dependent mechanisms in the perception of music and speech: Domain-specific transfer effects of elementary school music education. Journal of Research in Music Education.

[B54-jintelligence-10-00036] McMullen Erin, Saffran Jenny R. (2004). Music and language: A developmental comparison. Music Perception.

[B55-jintelligence-10-00036] Melby-Lervag Monica, Lyster Solveig-Alma H., Hulme Charles (2012). Phonological skills and their role in learning to read: A meta-analytic review. Psychological Bulletin.

[B56-jintelligence-10-00036] Miendlarzewska Ewa A., Trost Wiebke J. (2013). How musical training affects cognitive development: Rhythm, reward and other modulating variables. Frontiers in Neuroscience.

[B57-jintelligence-10-00036] Miksza Peter (2007). Music participation and socioeconomic status as correlates of change: A longitudinal analysis of academic achievement. Bulletin of the Council for Research in Music Education.

[B58-jintelligence-10-00036] Moreno Sylvain, Marques Carlos, Santos Andreia, Santos Manuela, Castro Sao L., Besson Mireille (2009). Musical training influences linguistic abilities in 8-year-old children: More evidence for brain plasticity. Cerebral Cortex.

[B59-jintelligence-10-00036] Moreno Sylvain, Friesen Deanna, Bialystok Ellen (2011). Effect of music training on promoting preliteracy skills: Preliminary causal evidence. Music Perception.

[B60-jintelligence-10-00036] Morgan Paul L., Farkas George, Hillemeier Marianne M., Maczuga Steven (2009). Risk factors for learning-related behavior problems at 24 months of age: Population-based estimates. Journal of Abnormal Child Psychology.

[B61-jintelligence-10-00036] Morrison Steven J. (1994). Music students and academic growth. Music Educators Journal.

[B62-jintelligence-10-00036] Moyeda Iris X. G., Gómez Ixtlixóchitl C., Flores María T. P. (2006). Implementing a musical program to promote preschool children’s vocabulary development. Early Childhood Research and Practice.

[B63-jintelligence-10-00036] Nagy József (2004). A szóolvasó készség fejlődésének kritériumorientált diagnosztikus feltérképezése [The diagnostic and criterion-referenced assessment of the development of the skill of word reading]. Magyar Pedagógia.

[B64-jintelligence-10-00036] Neisser Ulric, Boodoo Gwyneth, Bouchard Thomas J., Boykin A. Wade, Brody Nathan, Ceci Stephen J., Halpern Diane, Loehlin John C., Perloff Robert, Stenberg Robert J. (1996). Intelligence: Knowns and unknowns. American Psychologist.

[B65-jintelligence-10-00036] Nisbet Steven (1991). Mathematics and music. The Australian Mathematics Teacher.

[B66-jintelligence-10-00036] Okada Brooke M., Slevc L. Robert, Bunting Michael F., Novick Jared M., Dougherty Michael R., Engle Randall W. (2020). Musical training: Contributions to executive function. An Integrative Approach to Cognitive and Working Memory Training: Perspectives from Psychology, Neuroscience, and Human Development.

[B67-jintelligence-10-00036] Overy Katie, Nicolson Roderick I., Fawcett Angela J., Clarke Eric F. (2003). Dyslexia and music: Measuring musical timing skills. Dyslexia.

[B68-jintelligence-10-00036] Patel Aniruddh D., Rebuschat Patrick, Rohrmeier Martin, Hawkins John, Cross Ian (2012). Language, music, and the brain: A resource-sharing framework. Language and Music as Cognitive Systems.

[B69-jintelligence-10-00036] Peretz Isabelle (2009). Music, language and modularity framed in action. Psychologica Belgica.

[B70-jintelligence-10-00036] Portowitz Adena, Peppler Kylie A., Downton Mike (2014). In Harmony: A technology-based music education model to enhance musical understanding and general learning skills. International Journal of Music Education.

[B71-jintelligence-10-00036] Raven John, Raven J. C., Court John H. (1998). Manual for Raven’s Progressive Matrices and Vocabulary Scales.

[B72-jintelligence-10-00036] Santos-Luiz Carlos, Mónico Lisete S. M., Almeida Leandro S., Coimbra Daniela (2015). Exploring the long-term associations between adolescents’ music training and academic achievement. Musicae Scientiae.

[B73-jintelligence-10-00036] Schellenberg E. Glenn (2004). Music lessons enhance IQ. Psychological Science.

[B74-jintelligence-10-00036] Schellenberg E. Glenn (2006). Long-term positive associations between music lessons and IQ. Journal of Educational Psychology.

[B75-jintelligence-10-00036] Schellenberg E. Glenn (2011). Examining the association between music lessons and intelligence. British Journal of Psychology.

[B76-jintelligence-10-00036] Schellenberg Glenn, Weiss Michael W., Deutsch Diana (2013). Music and cognitive abilities. The Psychology of Music.

[B77-jintelligence-10-00036] Schneider Wolfgang, Näslund Jan C., Weinert Franz E., Schneider Wolfgang (1999). Impact of early phonological processing skills on reading and spelling in school: Evidence from the Munich longitudinal study. Individual Development from 3 to 12: Findings from the Munich Longitudinal Study.

[B78-jintelligence-10-00036] Shuter-Dyson Rosamund, Deutsch Diana (1999). Musical ability. The Psychology of Music.

[B79-jintelligence-10-00036] Sloboda John, Aiello Rita, Sloboda John (1994). Music performance: Expression and the development of exccellence. Musical Perceptions.

[B80-jintelligence-10-00036] Spelke Elizabeth, Asbury Carolyn, Rich Barbara (2008). Effects of music instruction on developing cognitive systems at the foundations of mathematics and science. Learning, Arts, and the Brain.

[B81-jintelligence-10-00036] Surján Noémi, Janurik Márta (2018). A zenei észlelés fejlettségének vizsgálata hagyományos és számítógépes tesztfelvétellel. [Examining the development of musical perception with traditional and computer-based testing.]. Gyermeknevelés.

[B82-jintelligence-10-00036] Tierney Adam, Kraus Nina, Merzenich Michael M., Nahum Mor, Vleet Thomas M. Van (2013a). Music training for the development of reading skills. Progress in Brain Research.

[B83-jintelligence-10-00036] Tierney Adam, Kraus Nina (2013b). The ability to tap to a beat relates to cognitive, linguistic, and perceptual skills. Brain and Language.

[B84-jintelligence-10-00036] Vaughn Kathryn (2000). Music and mathematics: Modest support for the oft-claimed relationship. Journal of Aesthetic Education.

[B85-jintelligence-10-00036] Wenger Win, Wenger Susan H. (1990). Training music sight-reading and perfect pitch in young children, as a way to enhance their intelligence. Journal of the Society for Accelerative Learning and Teaching.

[B86-jintelligence-10-00036] Wong Patrick C. M., Perrachione Tyler K. (2007). Learning pitch patterns in lexical identification by native English-speaking adults. Applied Psycholinguistics.

[B87-jintelligence-10-00036] Young Laura N., Cordes Sara, Winner Ellen (2014). Arts involments predicts academic achievement only when the child has a musical instrument. Educational Psychology: An Internationak Journal of Experimental Psychology.

[B88-jintelligence-10-00036] Zanutto Daniel R. (1997). The effect of instrumental music instruction on academic achievement (high school students). Doctoral dissertation, The University of California at Davis, Davis, CA, USA. Dissertation Abstracts International.

[B89-jintelligence-10-00036] Ziegler Johannes C., Goswami Usha (2005). Reading acquisition, developmental dyslexia, and skilled reading across languages: A psycholinguistic grain size theory. Psychological Bulletin.

[B90-jintelligence-10-00036] Holliman Andrew J., Wood Clare, Kieron Sheely (2010). Does speech rhythm sensitivity predict children’s reading ability 1 year later?. Journal of Educational Pychology.

[B91-jintelligence-10-00036] Law Lily (2012). Assessing and Understanding Individual Differences in Music Perception Abilities. Doctoral dissertation.

[B92-jintelligence-10-00036] Thackray R. (1969). An Investigation into Rhythmic Abilities.

